# A Proliferation-Inducing Ligand and B-Cell Activating Factor Are Upregulated in Patients with Essential Thrombocythemia

**DOI:** 10.3390/jcm11164663

**Published:** 2022-08-09

**Authors:** Lukasz Bolkun, Marlena Tynecka, Tomasz Wasiluk, Jaroslaw Piszcz, Aleksandra Starosz, Kamil Grubczak, Marcin Moniuszko, Andrzej Eljaszewicz

**Affiliations:** 1 Department of Haematology, Medical University of Bialystok, ul. M. Skłodowskiej-Curie 24A, 15-276 Bialystok, Poland; 2 Department of Regenerative Medicine and Immune Regulation, Medical University of Bialystok, ul. Waszyngtona 13, 15-269 Bialystok, Poland; 3 Regional Centre for Transfusion Medicine, Bialystok, ul. M. Skłodowskiej-Curie 23, 15-950 Bialystok, Poland; 4 Department of Allergology and Internal Medicine, Medical University of Bialystok, ul. M. Skłodowskiej-Curie 24A, 15-276 Bialystok, Poland

**Keywords:** APRIL, BAFF, megakaryocytopoiesis, essential thrombocythemia, CD34+ progenitor cells, megakaryocytes

## Abstract

A proliferation-inducing ligand (APRIL) and B-cell activating factor (BAFF) are cytokines belonging to the tumor necrosis factor family which play an essential role in B-cell maturation, differentiation, and survival. Recent evidence indicates their importance in hematological disorders; however, their function in essential thrombocytosis (ET) pathogenesis remains elusive. Therefore, we aimed to analyze the role of APRIL and BAFF in megakaryocytopoiesis in ET patients. We observed elevated levels of APRIL and BAFF in the plasma of ET patients compared with healthy controls, while no differences were found among patients with different JAK2(V617F) statuses. In addition, APRIL levels were positively associated with the number of platelets and WBC count. In the bone marrow, APRIL but not BAFF levels were higher in ET patients with the JAK2(V617F) mutation; however, JAK2(V617F)-negative patients showed slightly reduced levels of BAFF. In ET patients, we showed that the differentiation of CD34+ progenitor cells towards megakaryocytes induces the expression of both APRIL and BAFF. More importantly, APRIL neutralization significantly reduced platelet production. In conclusion, our data provide evidence that blocking APRIL signaling, which acts as an autocrine growth factor for terminal megakaryocytopoiesis, inhibits platelet production in ET patients, regardless of the status of JAK2(V617F) mutation.

## 1. Introduction

The regulation of megakaryocytopoiesis is a complex phenomenon that begins with the commitment of hematopoietic progenitor cells, which requires the coordinated activity of an array of soluble mediators, including cytokines and growth factors, to provide controlled cell proliferation, maturation, and differentiation [1]. It is well established that megakaryocyte (MKs) growth and platelet production in both physiologic and disease conditions are orchestrated by stem cell factors (SCFs), interleukin 1β (IL-1β), IL-6, and/or thrombopoietin (TPO), among others [2,3]. Increased platelet production is observed during severe inflammation, as the inflammatory environment promotes the formation of MKs in essential thrombocythemia: one of the chronic, BCR-ABL-negative, myeloproliferative neoplasms (MPN), characterized by an increased number of mature MKs and a sustained platelet count of above 450 × 10^9^ platelets/L [4].

The discovery of Janus-activated kinase 2 (JAK2)V617F, a gain-of-function JAK2 mutation, improved our understanding of MPN pathogenesis [5]. On the other hand, the mechanism by which this mutated JAK2 initiates deregulated signals in cells remains to be fully elucidated. It is believed that JAK2V617F requires interactions with cytokine receptors to elicit its transforming signal [5,6], which renders hematopoietic cells more sensitive to cytokine stimulation, probably influencing the phenotype of the disease [4,7]. Once activated, JAK family members lead to the transcriptional regulation of STAT target genes which regulate cell growth, death, and differentiation, among others [1,8].

Proinflammatory cytokines, such as tumor necrosis factor-α (TNF-α), are elevated in patients with MPN, but their contribution to disease pathogenesis remains elusive [9]. In addition, IL-1β and TNF-α have been shown to increase megakaryocytopoiesis through direct or indirect mechanisms, highlighting new issues regarding the potential physiopathologic role of plasma cytokines in MPN [9].

The B-cell activating factor (BAFF) and a proliferation-inducing ligand (APRIL) represent relatively newly discovered members of the tumor necrosis factor (TNF) family. BAFF and APRIL are produced as homotrimeric type II transmembrane proteins that may be cleaved to their unbound (soluble) forms by furin-like convertases [10]. Both ligands have been shown to act as main survival factors for immature, naive, and activated B-cells [10]. Their activity is mediated by direct interaction with shared receptors—namely, BCMA (B-cell maturation antigen) and TACI (transmembrane activator and CML interactor)—while BAFF is also explicitly recognized by BAFF receptor (BAFF-R). Strong evidence indicates their essential role in several hematopoietic disorders and malignancies [11]. BAFF and APRIL have been proposed to promote cancer cell survival, proliferation, and invasiveness, including breast cancer, multiple myeloma, and acute leukemia [12,13]. However, this effect was not demonstrated in lung cancer cells [14].

Interestingly, Bonci et al. showed that APRIL promotes the expansion of megakaryocytes from healthy cord blood-derived CD34+ progenitor cells [15]. It is, therefore, tempting to speculate that endogenous APRIL plays a role in MK growth and determines the amount of platelet production, suggesting a possible role in myeloproliferative neoplasms such as essential thrombocytosis (ET). Thus, we aimed to investigate the effects of BAFF and APRIL expression and function on megakaryopoiesis in ET patients.

## 2. Materials and Methods

### 2.1. Patients

Peripheral blood from 109 patients and bone marrow from 40 patients with newly diagnosed ET (following the World Health Organization classification system 2016) was provided by the Department of Hematology, Medical University of Bialystok. The detailed characteristics of patients enrolled in this study are summarized in Table 1. The samples were collected upon obtaining written informed consent, according to the rules and tenets of the recently revised Helsinki protocol (R-I-002/461/2018). All the patients were tested for JAK2(V617F) mutation. Patients who had CALR or MPL mutations, due to the small number of available samples, were excluded from the study. Furthermore, the patients were divided into two homogeneous groups according to their different biology: JAK2(V617F)-mutated patients and triple-negative patients. Indeed, there are several studies showing that triple-negative patients present a lower level of platelets or WBC and mostly have a low incidence of vascular events [16]. The patient’s median age at the time of sample collection was 59 years (interquartile range (IQR) 22–87 years). Seventy-five were female and thirty-nine were male. In the control group (age- and sex-matched), samples were obtained from 40 healthy volunteers (control).

### 2.2. Material Isolation

Freshly obtained EDTA-anticoagulated samples of blood and bone marrow were processed according to the standard operation procedures of the Medical University of Bialystok Biobank. Briefly, the blood was centrifuged at room temperature for 5 min at 400× *g* to separate plasma or bone marrow supernatant. Collected plasma and bone marrow supernatant was centrifuged at 4 °C for 5 min at 1200× *g* to remove residual cells. Aliquoted biofluids were biobanked in a −80 °C controlled environment.

Bone marrow mononuclear cells (BMMCs) were isolated by Histopaque density gradient centrifugation (Sigma, St. Louis, MO, USA) according to the manufacturer’s instructions. Briefly, freshly obtained bone marrow was diluted two times in PBS (Corning, New York, NY, USA) and placed on Histopaque (Sigma). The specimens were centrifuged for 25 min at room temperature, followed by interphase collection and washing in PBS for 5 min at 4 °C. Freshly isolated bone marrow mononuclear cells were counted, cryopreserved, and stored in LN_2_ for further use.

### 2.3. Immunoassay

BAFF, APRIL, IL-1β, IL-6, sTACl, and sBCMA were quantified in serum and bone marrow supernatant using commercially available ELISA sets (R&D Systems, Minneapolis, MN, USA) according to the manufacturer’s instructions. The detection range for used immunoassays was as follows: BAFF 39.1–2500 pg/mL, APRIL 31.3–2000 pg/mL, IL-1 β 3.91–250 pg/mL, IL-6 9.38–600 pg/mL, sTACI 93.8–6000 pg/mL, and sBCMA 31.3–2000 pg/mL. The samples were analyzed using an automatic light absorption reader, Ledetec. The results were calculated according to the standard curve in MicroWin2000 software (Baton Rouge, LA, USA).

### 2.4. Cell Sorting

**Figure 1:**
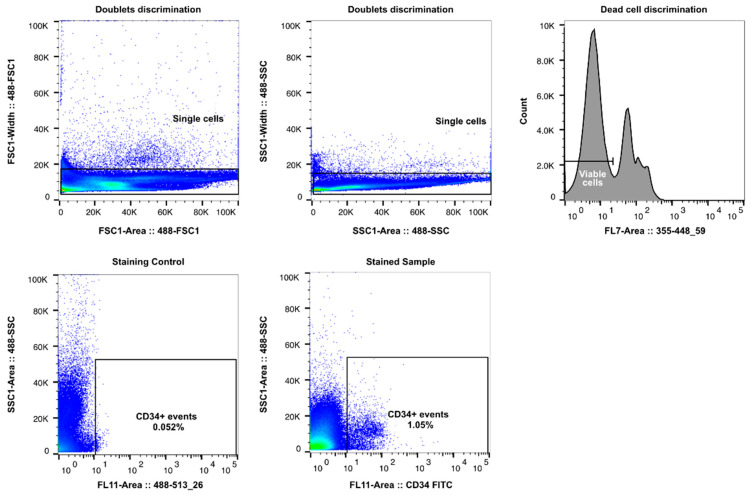
Gating strategy for CD34+ cell sorting. Representative flow cytometry plots representing gating strategy used for CD34+ hematopoietic stem cell sorting from bone marrow. First, doublet discrimination was performed according to forward (FSC, Bonn, Germany) and side (SSC, New Delhi, India) scatter. Next, the Boolean single event gate (“AND” gate) was visualized on a histogram for dead cell discrimination. Viable CD34+ cells were sorted within the CD34+ cells based on single-cell purity. The CD34+ gate was set up according to fluorescence minus one control (Staining Control).

Bone marrow mononuclear cells were thawed, washed, and resuspended in X VIVO-10 (Thermo Fisher, Waltham, MA, USA) culture medium and incubated for resting (2–3 h at 37 °C, 5% CO_2_). Next, the cells were washed in phosphate-buffered saline (PBS, Arlington County, VA, USA; Corning) and stained with Zombie UV Viability Dye (Biolegend, San Diogo, CA, USA), according to manufacturer’s instructions. Finally, the cells were stained with mouse anti-human anti-CD34 FITC conjugated monoclonal antibody (Biologend, clone 561). Hematopoietic stem cells, defined as single/viable/CD34+ events (Figure 1), were sorted using the MoFlo Astrios Cell Sorting System (Beckman Coulter, Brea, CA, USA), using single-cell purity into X VIVO-10 medium (Thermo Fisher). Sorted cells were immediately used for differentiation experiments.

### 2.5. Hematopoietic Stem Cell Differentiation

Freshly sorted CD34+ hematopoietic stem cells were stimulated in vitro with rhTPO (Recombinant Human Thrombopoietin, R and D Biosystems, Minneapolis, MN, USA) in the presence or absence of TACI: Fc (recombinant human fusion receptor used for blocking of growth factor function; ENZO) in X VIVO-10 medium (Thermo Fisher, Waltham, MA, USA) in 96-well plates (Corning) [15]. The medium was changed every second day. After seven days of stimulation, the culture supernatant and cells were harvested for further analysis. The number of platelets in the cell culture medium was assessed by using an automated reader (Sysmex XN300), and by two experienced hematologists independently.

### 2.6. qPCR

Total RNA isolation was performed using the RNeasy Micro kit (QIAGEN), according to the manufacturer’s instructions. Next, mRNA was reverse-transcribed using a high-capacity cDNA reverse-transcription kit (Thermo Fisher). Expression levels of BAFF, APRIL, and their receptors (Thermo Fisher) were quantified using commercially available TaqMan assays (Thermo Fisher) on the StepOnePlus system (Life Tehcnologies, Hong Kong, China). The relative expressions of BAFF (Hs00198106_m1) and APRIL (Hs00601664_g1) were calculated and normalized to GAPDH (PN4351370) expression.

### 2.7. Statistics

Statistical analysis was performed using GraphPad Prism 8 Software (GraphPad Software, San Diego, CA, USA). The Mann–Whitney U test or Wilcoxon matched pairs test was used. The Spearman’s rank correlation coefficient was used to examine correlations. Statistically significant results were identified at *p* < 0.05. The data were presented as median ± interquartile range.

## 3. Results

**Figure 2:**
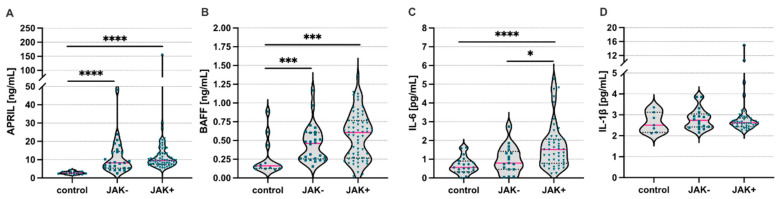
Systemic levels of A proliferation-inducing ligand (APRIL), B cell activating factor (BAFF), Interleukin (IL)-6, and IL-1β in the essential thrombocytosis patients with or without JAK2(V617F) mutation. Summary of analyses of (**A**) APRIL, (**B**) BAFF, (**C**) IL-6, and (**D**) IL-1beta levels in serum of ET patients with different JAK2(V617F) status and healthy controls. Control—healthy volunteers; JAK-/+ absence/presence of the JAK2(V617F) mutation; Mann–Whitney U test was used; * *p* < 0.05; *** *p* < 0.001; **** *p* < 0.0001.

First, we analyzed systemic levels of APRIL and BAFF in ET patients (with or without JAK2V617F mutation) and compared the results to matched healthy volunteers. We found significantly higher concentrations of APRIL and BAFF in ET patients when compared to healthy controls, regardless of the presence of the JAK2(V617F) mutation (Figure 2A,B, respectively). Moreover, we found elevated levels of IL-6 in patients with the JAK2(V617F) mutation when compared to both control subjects and ET patients without mutation (Figure 2C). However, we found no differences in the levels of IL-1β among the analyzed groups (Figure 2D). Additionally, we observed a positive, but weak, correlation between APRIL concentrations and the number of platelets (r = 0.257; *p* = 0.044) and WBC counts (r = 0.3025; *p* = 0.017).

**Figure 3:**
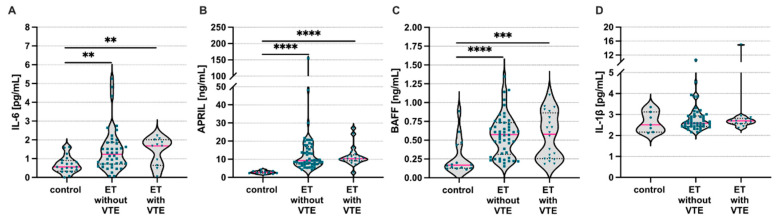
Systemic levels of interleukin (IL)-6, a proliferation-inducing ligand (APRIL), B-cell activating factor (BAFF), and IL-1β in the essential thrombocytosis patients with or without venous thromboembolism. Systemic levels of (**A**) IL-6, (**B**) APRIL, (**C**) BAFF, and (**D**) IL-1beta among patients with or without venous thromboembolism (VTE) and control healthy individuals. Control—healthy volunteers; ET—essential thrombocytosis patients; VTE—venous thromboembolism; Mann–Whitney U test was used; ** *p* < 0.01; *** *p* < 0.001; **** *p* < 0.0001.

In our study among 109 patients, 18% (20 individuals) were presenting with thrombosis (VTE). As a result, we next aimed to evaluate systemic levels of analyzed inflammatory mediators in patients with and without VTE. We found higher levels of IL-6 (Figure 3A), APRIL (Figure 3B), and BAFF (Figure 3C), but not IL-1β (Figure 3D), in both groups of patients compared to control subjects. Surprisingly, we found no differences comparing patients with and without VTE. However, we observed elevated IL-6 levels in patients with VTE carrying JAK2(V617F) mutation compared to the patients without mutation (1.85; 1.131–2.053 vs. 1.49; 0.624–1.903, *p* = 0.01, respectively).

**Figure 4:**
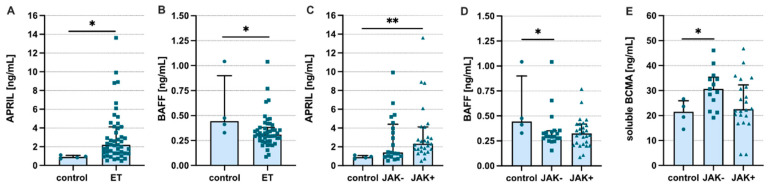
Levels of a proliferation-inducing ligand (APRIL) and B-cell activating factor (BAFF) in bone marrow supernatants. Summary of analyses of (**A**) APRIL and (**B**) BAFF in the bone marrow of ET patients and matched healthy controls. Levels of (**C**) APRIL, (**D**) BAFF, and (**E**) sBCMA among patients with different JAK2(V617F) status and healthy controls. Control-healthy volunteers; JAK-/+ absence/presence of the JAK2(V617F) mutation; ET-essential thrombocytosis patients; Mann-Whitney U test was used; * *p* < 0.05; ** *p* < 0.01.

Having found a systemic increase in BAFF and APRIL levels in patients with ET, we next analyzed concentrations of the cytokines mentioned above in the bone marrow supernatant. We found elevated levels of APRIL in ET patients when compared to healthy controls (Figure 4A). Surprisingly, however, BAFF levels were significantly lower in the analyzed patients (Figure 4B). A higher level of APRIL was observed in patients with JAK2(V617F) mutation (Figure 4C). In contrast, significantly lower levels of BAFF were observed only in ET patients without JAK2(V617F) mutation, while in patients with the mutation, only a trend (at the threshold of statistical significance) was observed (Figure 4D). Moreover, we found increased concentrations of APRIL and BAFF soluble receptors—namely, sBCMA (Figure 4E) but not sTACI (data not shown)—in the bone marrow of patients without the JAK2(V617F) mutation. Moreover, the levels of IL-6 and IL-1β in the bone marrow did not reach the detection limit of the used assays (data not shown).

**Figure 5:**
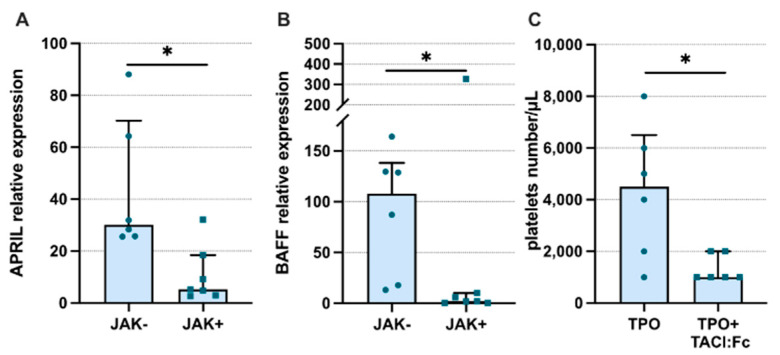
A proliferation-inducing ligand (APRIL) and B-cell activating factor (BAFF) expression levels are upregulated during TPO-induced megakaryocytopoiesis. (**A**) APRIL and (**B**) BAFF expression in bone marrow-derived CD34+ progenitor cells in the course of TPO-induced megakaryocyte differentiation. The PCR results were presented as relative expression level (2-ΔcT). (**C**) The effect of APRIL and BAFF neutralization using TACI:Fc fusion protein on platelet production in the course of TPO-induced bone marrow-derived CD34+ progenitor cell in vitro differentiation. JAK-/+ absence/presence of the JAK2(V617F) mutation; TPO—thrombopoietin; TACI: FC—recombinant human TACI-Fc fusion protein; Mann-Whitney U Test was used; * *p* < 0.05.

Finally, we analyzed whether the observed elevated systemic and local levels of APRIL in ET patients may play a direct role in pathological megakaryopoiesis. Using bone marrow-derived CD34+ cells from ET patients, we confirmed the previous observations of Bonci et al. that both APRIL and BAFF mRNA could not be detected in freshly isolated progenitor cells (data not shown) [15]. Next, we found that the expression of both ligands is induced during MK maturation (Figure 5A,B, respectively). Interestingly, APRIL and BAFF expression was significantly elevated in cells without JAK2(V617F) mutation compared to in unmutated counterparts. Furthermore, we could not detect TACI and BCMA expression (data not shown) in both freshly isolated and maturating CD34+ progenitor cells. In order to determine whether endogenous APRIL contributes to the megakaryopoiesis process, CD34+ progenitor cells were differentiated in the presence of soluble human recombinant TACI-Fc fusion protein to enable the blocking of APRIL function. We found that APRIL neutralization resulted in a significant decrease in platelet production (Figure 5C). Thus, we presented the first functional evidence of the contribution of autocrine APRIL production to megakaryocytopoiesis.

## 4. Discussion

Regardless of constitutive activation of JAK/STAT signaling, due to somatic mutations in JAK2(V617F), calreticulin or the thrombopoietin receptor, it is hypothesized that other non-genetic factors, including inflammatory mediators, are involved in the pathogenesis of ET. A growing body of evidence indicates a substantial role of inflammatory cytokines (such as IL-1β and IL-6) and growth factors in the control of MK differentiation, growth, and platelet production. These promegakaryocytic factors may act through direct interaction with MKs, or by indirectly stimulating cells that form a hematopoietic niche in the bone marrow [1,17,18]. More importantly, it has been shown that MKs can support their own growth and differentiation program in an autocrine manner by the release of platelet-derived growth factor and von Willebrand factor [19].

APRIL and BAFF are mainly produced by myeloid cells, including monocytes/macrophages, dendritic cells, and granulocytes. In addition, they possess high immune-modulatory properties and may interact with both innate and adaptive cellular repertoires [20,21,22,23,24]. In fact, BAFF and APRIL have been shown to play a crucial role in B-cell development, maturation, differentiation, and antibody isotype switching. Furthermore, it has been proposed that both APRIL and BAFF may play a role in the differentiation and maturation of other hematopoietic cells in bone marrow, including megakaryocytic cells [15]. Bonci et al. showed, by using cord-blood-derived CD34+ cells, that APRIL is upregulated during the proliferative phase of megakaryocytic cell differentiation, and exogenous APRIL increases the megakaryocytic cell growth [15]. However, it should be emphasized that, to date, the direct or indirect influence of APRIL and BAFF has never been described in the pathogenesis of ET, particularly in the process of megakaryopoiesis.

Our study evaluated systemic and bone marrow concentrations of APRIL and BAFF and their receptors in ET patients, and showed their significantly higher concentrations in peripheral blood, regardless of the presence of the JAK2(V617F) mutation. However, we found that with APRIL, but not BAFF, the level was elevated in the bone marrow of ET patients, primarily in those presenting with JAK2(V617F) mutation. Moreover, we established a positive association between APRIL concentration and platelet and WBC counts; both are referred to as risk factors for thrombosis according to the International Prognostic Score for ET [25]. Notably, granulocytes and monocytes from ET patients express higher BAFF levels than those of healthy adults. This explains the higher systemic levels of BAFF in our study. On the other hand, BAFF was shown to increase transcription and the release of IL-6 in monocytes/macrophages and B-cells [20]. Moreover, BAFF enhances Toll-like receptor 4 (TLR4) expression in B-cells [26]. Upon TLR-4 stimulation, B-cells release high levels of inflammatory cytokines, including IL-6. Interestingly, IL-6 and IL-1β have promoted MK growth and platelet production [27]. IL-1β enhances nuclear factor E2 (NF-E2) expression in MKs [28], which has been found to facilitate the proliferation and differentiation of hematopoietic progenitors [29]. In contrast, IL-6 contributes to thrombopoiesis by activating TPO gene transcription and its increased release [30]. Here, we evaluated systemic and local (bone marrow) concentrations of IL-6 and IL-1β, and showed elevated levels of IL-6 in serum of JAK2(V617F)-positive patients compared to the control and patients without the mutation. Moreover, we found significantly higher concentrations of IL-6 in patients with VTE carrying the JAK2 (V617F) mutation, compared to the mutation-negative patients. In fact, inflammation is associated with one of the significant complications of MPNs, namely, thromboembolism. MPN patients show an abnormal expression of integrin CD11b in leukocytes and *p*-selectin receptor (CD62p) in platelets. This overexpression led to an enhanced formation of platelet–leukocyte complexes [31]. Finally, the increased activation of the hemostatic system, manifested by elevated levels of markers of thrombosis, such as D-dimers, von Willebrand factor, and prothrombin fragments, suggests that, in addition to hypercellularity, abnormalities in the coagulation pathway contribute to the prothrombotic state in MPNs, including ET patients [32].

The essential role of APRIL in megakaryocyte differentiation and platelet production in cord-blood-derived CD34+ cells has been suggested [15]. However, in our study, it remained elusive whether the observed elevated levels of APRIL in the bone marrow play a role in the pathology of ET. Thus, we used an in vitro differentiation assay with APRIL neutralization to assess platelet production and cell phenotype. Unfortunately, due to the low number of CD34+ cells in the bone marrow and the limited amount of material that can be acquired from the patients, the expression of APRIL and BAFF was evaluated at baseline (in freshly isolated cells before differentiation) and on day seven of megakaryocytic cells differentiation induced by TPO. As in other studies, APRIL and BAFF mRNA were undetectable at baseline; however, TPO-induced differentiation induced their expression. Interestingly, the expression of both analyzed TNF family members was elevated in differentiating cells from ET JAK2(V617F)-negative patients compared to JAK2(V617F)-positive counterparts. In contrast, we could not detect TACI or BCMA expression during megakaryocytic differentiation, suggesting that APRIL binding to MKs depends on the presence of an as-yet undefined pathway. Several epithelial cell lines can proliferate more rapidly in response to APRIL, even though they do not express BCMA or TACI [33,34]. This observation was extended with a subcutaneous tumor model of HT29 and A459 cell lines in which tumor growth was significantly reduced and nearly prevented by the administration of soluble BCMA:Fc fusion proteins [35]. This pathway may involve APRIL interaction with proteoglycans, mediated by heparan sulfate (HS) side chains and inhibited by the heparin [36,37]. APRIL binds HS proteoglycans via the lysine-rich region in the N-terminal part, leaving the TNF-like region available to interact with other receptors [38,39].

To determine whether the production of endogenous APRIL contributes to the megakaryopoiesis process, CD34+ cells isolated from bone marrow patients with ET (with and without JAK2 (V617F) mutation) were grown in MK culture in the presence of soluble human recombinant TACI:Fc fusion protein, which neutralizes APRIL activity [15]. In fact, we presented here, direct evidence that APRIL neutralization decreases platelet production, confirming the contribution of autocrine APRIL production to megakaryocytopoiesis, regardless of the presence of the JAK2(V617F) mutation. Moreover, the data presented here indicate that APRIL acts as an autocrine growth factor for megakaryocytic cells with and without JAK2(V617F) mutation, offering a potential new point for biological therapies. The therapeutic potential and efficiency of APRIL neutralization have been proved in several hematological malignancies. APRIL neutralization with antibodies and BCMA downregulation significantly decrease myeloma cell viability and colony formation [40]. However, BCMA control has been retained as a compelling therapeutic target in myeloma, with a limited risk of off-tissue toxicity [41]. In 2013, the first report of an anti-BCMA CAR-expressing T (CAR-T) cell was published [42], promoting BCMA as a target for multiple myeloma treatment.

## 5. Conclusions

In summary, the contribution of APRIL to megakaryocytopoiesis appears to be due to the immediate increase in MK maturation, and the process itself seems to be independent of the presence of (JAK2)V617F mutation. More importantly, we showed direct evidence that APRIL may act as an autocrine growth factor for megakaryocytes. However, further studies are needed to elucidate the observed phenomenon. We believe that our research opens new possibilities for targeted APRIL-oriented therapy in ET.

## Figures and Tables

**Table 1:** Demographic and clinical characteristics of the ET patients and healthy volunteers.

Parameter	All Donors			ET Patients		
	ET (*n* = 109)	Control (*n* = 40)	*p*	JAK2+ (*n* = 75)	JAK2- (*n* = 34)	*p*
age (min–max)	59 (23–87)	58 (23–85)	*p* = 0.9	58 (23–84)	59 (25–87)	*p* = 0.88
female/male	75/39	25/15	*p* = 0.9	52/27	27/12	*p* = 0.79
RBC (×10^6^/µL)	4.85 (4.4–5.17)	4.12 (3.9–4.45)	*p* = 0.09	4.97 (4.6–5.29)	4.725 (4.14–5.07)	*p* = 0.22
HGB (g/dL)	14.4 (13.1–15.3)	14.1 (12.3–15.4)	*p* = 0.2	14.7 (13.2–15.5)	13.8 (12.8–14.9)	*p* = 0.1
HCT (%)	43.5 (39.45–47)	42 (39.1–45.1)	*p* = 0.09	44.1 (39.9–47.9)	40.8 (37.7–45.0)	*p* = 0.02
WBC (×10^3^/µL)	9.71 (7.9–11.83)	7.12 (5.1–9.2)	*p* = 0.01	10.32 (8.3–12.53)	8.9 (6.46–10.29)	*p* = 0.002
PLT (×10^3^/µL))	828 (678.5–994)	255 (166–340)	*p <* 0.01	876 (712–1072)	764.5 (610–941.3)	*p* = 0.06
history of thrombosis (patients)	*n* = 20	-	-	*n* = 13	*n* = 7	-
risk stratification for thrombosis (IPSET-t)						
low	*n* = 44	-	-	*n* = 22	*n* = 20	-
intermediate	*n* = 53	-	-	*n* = 30	*n* = 23	-
high	*n* = 17	-	-	*n* = 10	*n* = 7	-

ET—essential thrombocytosis; JAK2+, patients with JAK2 V617F mutations; PLT—platelets’ WBC—white blood cells; HCT—hematocrit; Hgb—hemoglobin; RBC—red blood cells; IP—SET-t; International Prognostic Score of thrombosis in World Health Organization—essential thrombocythemia. Data are presented as medians (IQR).

## Data Availability

The datasets used and/or analyzed during the current study are available from the corresponding author on reasonable request.
